# Depletion of Neutrophils Promotes the Resolution of Pulmonary Inflammation and Fibrosis in Mice Infected with *Paracoccidioides brasiliensis*

**DOI:** 10.1371/journal.pone.0163985

**Published:** 2016-09-30

**Authors:** Juan David Puerta-Arias, Paula Andrea Pino-Tamayo, Julián Camilo Arango, Ángel González

**Affiliations:** 1 Medical and Experimental Mycology Unit, Corporación para Investigaciones Biológicas (CIB), Medellín, Colombia; 2 School of Microbiology, Universidad de Antioquia, Medellín, Colombia; 3 Basic and Applied Microbiology Research Group (MICROBA), Universidad de Antioquia, Medellín, Colombia; University of Minnesota, UNITED STATES

## Abstract

Chronic stages of paracoccidioidomycosis (PCM) are characterized by granulomatous lesions which promote the development of pulmonary fibrosis leading to the loss of respiratory function in 50% of patients; in addition, it has been observed that neutrophils predominate during these chronic stages of *P*. *brasiliensis* infection. The goal of this study was to evaluate the role of the neutrophil during the chronic stages of experimental pulmonary PCM and during the fibrosis development and tissue repair using a monoclonal specific to this phagocytic cell. Male BALB/c mice were inoculated intranasally with 1.5x10^6^ *P*. *brasiliensis* yeast cells. A monoclonal antibody specific to neutrophils was administered at 4 weeks post-inoculation followed by doses every 48h during two weeks. Mice were sacrificed at 8 and 12 weeks post-inoculation to assess cellularity, fungal load, cytokine/chemokine levels, histopathological analysis, collagen and expression of genes related to fibrosis development. Depletion of neutrophils was associated with a significant decrease in the number of eosinophils, dendritic cells, B cells, CD4-T cells, MDSCs and Treg cells, fungal load and levels of most of the pro-inflammatory cytokines/chemokines evaluated, including IL-17, TNF-α and TGF-β1. Recovery of lung architecture was also associated with reduced levels of collagen, high expression of TGF-β3, matrix metalloproteinase (MMP)-12 and -14, and decreased expression of tissue inhibitor metalloproteinase (TIMP)-2, and MMP-8. Depletion of neutrophils might attenuate lung fibrosis and inflammation through down-regulating TGF-β1, TNF-α, IL-17, MMP-8 and TIMP-2. These results suggest that neutrophil could be considered as a therapeutic target in pulmonary fibrosis induced by *P*. *brasiliensis*.

## Introduction

Pulmonary fibrosis is the final outcome of a progressive, uncontrolled and irreversible process of tissue regeneration triggered by exposure to multiple agents such as environmental toxins, radio/chemotherapy and some pathogens, including dimorphic fungi such as *Paracoccidiodes* spp., the causal agent of paracoccidioidomycosis (PCM) [1,2].

PCM is a systemic fungal infection of great importance in Latin America, mainly in Brazil, Colombia, and Venezuela. It is estimated that 10 million people are infected with this pathogen, of which only about 1–2% will develop the mycosis [3,4], mostly with chronic and progressive form of the disease (90%) [5]. This chronic stage of PCM is characterized by a granulomatous inflammatory response and progressive damage in the lung tissue as a response to the fungus, which remains even after treatment and promotes the pulmonary fibrosis with loss of respiratory function in 50% of the patients [6].

Multiple studies have demonstrated the relevance of neutrophils in the pathogenesis of PCM, especially during the early stages of infection [7,8]. Recently, it has been suggested that neutrophils may modulate the innate and adaptive immune response against *P*. *brasiliensis* infection through the production of cytokines and lipid mediators that lead the immune system toward a protective response mediated by a Th1-like pattern [9,10]. On the other hand, Pina *et al*. [11] using an antibody-mediated neutrophil depletion strategy in an animal model of pulmonary PCM demonstrated that functionality of neutrophils was dependent on the host genetic pattern; thus, when compared resistant (A/J) and susceptible (B10.A) mice to *P*. *brasiliensis* infection and treated with an anti-neutrophil monoclonal antibody (mAb) anti-Gr1 (clone RB6-8C5), susceptible animals showed a decrease in survival time, increase on fungal burden in the lung, liver, and spleen as well as higher levels of interleukin (IL)-4, IL-12, hepatic cytokines and synthesis of antibodies associated with Th1 and Th2 profiles; in contrast, resistant mice treated with the mAb showed higher levels of IL-12, GM-CSF and Th1-associated antibodies with diminished levels of hepatic cytokines, thus evidencing the importance of neutrophils according to the host genetic background [11]. More recently, and using an intermediate susceptible mouse model to *P*. *brasiliensis* infection, we demonstrated that neutrophils are essential for protection and also important to regulate immunopathology in PCM during the early stages of infection [12].

Actually, it has not been demonstrated how the neutrophil exerts its role during the development of the granulomatous response in *P*. *brasiliensis* infection and fibrotic outcome in PCM. Therefore, we aimed to determine the role of neutrophils during the chronic stage of PCM in BALB/c strain mice which have intermediate susceptibility to *P*. *brasiliensis* infection [13] treated with the anti-neutrophils mAb anti-Ly6G (clone 1A8). Our results showed that neutrophil depletion during these chronic stages of *P*. *brasiliensis* infection was associated with a decrease in the granulomatous inflammatory response and fungal load as well as with recovery of lung architecture with attenuation of pulmonary fibrosis, thus indicating a detrimental role of these phagocytic cells in the chronic stages of PCM and during development of fibrosis process.

## Materials and Methods

### Mice

BALB/c male mice of eight week-old were obtained from the breeding colony maintained at Corporación para Investigaciones Biológicas, CIB (Medellín, Colombia). Two experimental groups of mice were gathered which consisted of infected or non-infected control mice. Mice from both groups were split into sub-groups in order to undergo the following treatment regimens for each evaluation time: untreated, isotype-treated and neutrophil-depleted animals.

### Ethics statement

This study was carried out following the Colombian (Law 84/1989, Resolution No. 8430/1993), European Union, and Canadian Council on Animal Care regulations. The protocol was approved by the Institutional Ethics Committee of the Corporación para Investigaciones Biológicas (# PRE00501023085, Acta No.92).

### *Paracoccidioides brasiliensis* yeasts

In this study, a highly virulent *P*. *brasiliensis* isolate (Pb18) was used. Yeast cells were subcultured every week on slant agar tubes with supplemented medium; Sabouraud Dextrose Agar (Difco Laboratories, Detroit, MI, USA), 0.14% L-asparagine (Sigma-Aldrich, Saint Louis, MO, USA), 0.01% thiamine hydrochloride (Sigma-Aldrich, Saint Louis, MO, USA) and 100 U/ml Penicillin– 100 μg/ml Streptomycin (GIBCO Invitrogen Corporation, Carlsbad, CA, USA), and incubated at 36°C in 5% CO_2_. Yeast cells were transferred into 100 ml supplemented medium as previously described and incubated during 4 days at 36°C at 150 rpm. Then, yeast suspension was pelleted by centrifugation (1400g, 10°C for 10 min), washed twice with 1X PBS, pH = 7.4 (GIBCO Invitrogen Corporation, Carlsbad, CA, USA) and passed 20 times through a 21G x 1.5 inch needle using a 10 ml syringe in order to obtain between 70 to 80% individual yeast cells. The yeast suspension was allowed to settle during 10 min and supernatant was transferred to a new tube. Cell suspension was concentrated by centrifugation and counted in a hemocytometer to determine the percentage of individual yeast cells and their viability using Janus Green vital dye (Acros Organics, Geel, Belgium). The number of yeast was adjusted to 1.5x10^6^ cells contained in 60μl to infect the mice.

### *Paracocccidioides brasiliensis* infection

Intranasal inoculation of cell suspension previously described (1.5x10^6^ yeast cells in 60μl PBS) was performed in anesthetized animals by intramuscular injection of 50μl Ketamine (80mg/kg) (Laboratorios Biosano, Santiago, Chile)–Xylazine (8mg/kg) (Bayer S.A., Bogotá, Colombia) solution. The total inoculum was split into two equal doses instilled intranasally within a 5–10 min period. Non-infected (control) mice were inoculated with 60μl of PBS.

### Depletion of neutrophils

Mice were injected intraperitoneally with 200μl solution containing 200μg of the mAb anti-Ly6G, clone 1A8 (Bio X Cell, West Lebanon, NH, USA). Control mice were injected with an equivalent amount of an isotype control IgG2a, clone 2A3 (Bio X Cell, West Lebanon, NH, USA). During the chronic phase of the disease, mAb treatment was started at 4 or 8 weeks post-inoculation (with PBS or *P*. *brasiliensis*), followed by additional doses every 48h over a period of two weeks.

### Colony forming units determination (CFU)

Mice groups were sacrificed at 12 weeks post-infection after undergoing the corresponding treatment. Lungs, livers, and spleens were removed, weighed and homogenized in 2ml sterile 1X PBS-100 U/ml Penicillin-100 μg/ml Streptomycin solution using a gentleMACS Dissociator (Miltenyi Biotec, Teterow, Germany). Homogeneous suspensions of those tissues were diluted (1:10, 1:100 and 1:1000) and 0.5ml of each dilution was plated on Petri dishes added with Brain Heart Infusion (BHI) agar medium (BD BBL, Franklin Lakes, NJ, USA) supplemented with 0,5% D-(+)-Glucose (Sigma-Aldrich, Saint Louis, MO, USA), 4% horse serum (GIBCO Invitrogen Corporation, Carlsbad, CA, USA) previously heating inactivated at 56°C for 30 min, and 300μM EDTA (Sigma-Aldrich, Saint Louis, MO, USA), followed by incubation at 36°C, 5% CO_2_. CFU counts were performed during the first 11 days after plating homogeneous suspensions. In order to determine the normalized CFU count, it was applied the following formula: CFU/g of tissue = total number of colonies multiplied by the total dilution factor which was obtained by multiplying dilution factors for each of the following parameters: homogenized tissue (total volume of homogenized tissue/g of tissue), volume plated on Petri dishes (0.5ml) and diluted homogeneous suspension plated on Petri dishes. Data was transformed into Log_10_ CFU/g of tissue.

### Determination of cytokines and chemokines

Mice were sacrificed at 12 weeks post-infection after undergoing the corresponding treatment. Lungs were removed and homogenized as described previously, homogeneous suspension was added with protease inhibitor cocktail (Roche Applied Science, Mannheim, Germany), mixed and centrifuged (3,000 rpm, 4°C for 5 min). Aliquots of supernatants were stored at 70°C until being used. The following cytokines and chemokines were determined from those supernatants: CC chemokine ligand (CCL)-2 (monocyte chemoattractant protein 1, MCP-1), CCL-3 (macrophage-inflammatory protein 1α, MIP-1α), CCL-4 (MIP-1β), CCL-5 (RANTES), CCL-11 (Eotaxin), CXC chemokine ligand (CXCL)-1 (keratinocyte chemoattractant, KC), CXCL-2 (MIP-2), CXCL-5 (lipopolysaccharide induced CXC chemokine, LIX), CXCL-9 (MG), CXCL-10 (interferon-inducible protein-10, IP-10), granulocyte colony-stimulating factor (G-CSF), monocyte colony-stimulating factor (M-CSF), GM-CSF, IFNγ, IL-1α, IL-1β, IL-2, IL-3, IL-4, IL-5, IL-6, IL-7, IL-9, IL-10, IL-12(p40), IL-12(p70), IL-13, IL-15, IL-17, TNFα, leukemia inhibitory factor (LIF), and vascular endothelial growth factor (VEGF). Determination of these molecules was performed by a multiplex assay using a commercial kit and the Luminex 200 system (EMD Millipore, Billerica, MA, USA).

### Flow cytometry

Lungs from mice were removed and homogenized using 40 and 70μm sterile cell strainers (Thermo Fisher Scientific Inc, Waltham, MA, USA) in RPMI plus 1% FBS (Sigma-Aldrich, Saint Louis, MO, USA) previously heating inactivated at 56°C for 30 min. Cell suspension was pelleted by centrifugation (1500 rpm, 10°C for 10 min), red blood cells were lysed by ACK Lysing Buffer for 3–5 min (GIBCO Invitrogen Corporation, Carlsbad, CA, USA), and RPMI plus 1% FBS was added to stop the reaction and wash the cells. Then, cell suspension was pelleted again, resuspended in RPMI plus 10% FBS and counted in a hemocytometer. The cell suspension was divided among several wells on a 96-well plate in order to determine the following cell populations: neutrophils, eosinophils, DCs, macrophages, B cells, CD8 T, CD4 T and NK cells. Fc receptors were blocked using a purified rat anti-mouse CD16/CD32 (BD Pharmingen, San Diego, CA, USA) and cells were immunostained in FACS buffer (1X PBS/0.5% FBS) using fluorescent mAbs against murine surface molecules and isotype controls as follows: Fluorescein isothiocyanate (FITC)-Rat IgG_2b_κ (A95-1), Phycoerythrin (PE)-Rat IgG_2a_κ (R35-95), Allophycocyanin (APC)-Rat IgG_1_κ (R3-34), V450-Rat IgG_2b_κ (A95-1), APC-Cy7-Rat IgG_1_κ (R3-34), FITC-anti-CD45 (30-F11), APC-anti-Ly-6G (1A8), APC-Cy7-anti-Ly-6G and Ly-6C (RB6-8C5), PE-anti-CD11b (M1/70), APC-anti-CD11c (HL3), PE-anti-Siglec-F (E50-2440), PE-anti-Mac-3 (M3/84), PE-anti-CD23 (B3B4), APC-anti-IgM (II-41), PE-anti-CD3 (17A2), PerCP-anti-CD8a (53–6.7), PerCP-Cy5.5-anti-CD4 (RM4-5), PE-anti-CD25 (PC61), APC-anti-NK-1.1 (PK136), PE-Cy7-anti-IFNγ (XMG1.2), PE-Cy7-anti-IL-4 (11B11), APC-Cy7-anti-IL-17a (TC11-18H10), V450-anti-IL-10 (JES5-16E3), and Alexa Fluor 647-anti-FoxP3 (MF23) (BD Pharmingen, San Diego, CA, USA), PE-anti-IL-22 (Poly5164) (Biolegend). For Treg cells and intracellular cytokines analysis, cells were treated with Cytofix/Cytoperm^™^ solution—Perm/Wash^™^ solution (BD Pharmingen, San Diego, CA, USA) and intracellular staining. After washing cell suspensions in FACS buffer, they were fixed with FACS buffer/1% PFA (Carlo Erba, Barcelona, Spain). Analysis was determined using a FACS Canto II system (BD Biosciences, San Jose, CA, USA) and FlowJo V10 software (FlowJo, LLC, Data Analysis software, Ashland, OR, USA). Cell populations were analyzed as follows: (a) cell events in region 1 (R1) were gated by forward scatter versus side scatter areas; (b) CD45^+^ events were gated from R1 by side scatter area versus CD45 staining to establish the R2 region, from which (c) cell events were gated according to the specific surface marker to determine the specific cell subpopulation and specific intracellular markers. The total number of leukocytes was obtained multiplying the counted of cell suspension by the percentage of CD45^+^ cells. The number of each leukocyte subpopulation was determined by multiplying the percentage of each gated subpopulation by the total number of leukocytes (CD45^+^ population). Leukocytes subpopulations were defined as follows: neutrophils: CD45^+^/CD11b^+^/Gr1^+^/Ly6G^+^, eosinophils: CD45^+^/CD11c^-^/SiglecF^+^, myeloid-derived suppressor cells: CD45^+^/CD11b^+^/Gr1^+^/Ly6G^-^, alveolar macrophages: CD45^+^/CD11c^+^/CD11b^low/med^, DCs: CD45^+^/CD11c^+^/CD11b^high^, tissue macrophages: CD45^+^/CD11c^+^/Mac3^+^, B cells: CD45^+^/CD23^+^/IgM^+^, CD8 T cells: CD45^+^/CD3^+^/CD8^+^, CD4 T cells: CD45^+^/CD3^+^/CD4^+^, NK cells: CD45^+^/CD3^-^/NK1.1^+^, CD4 T helper-1 cells: CD45^+^/CD4^+^/IFNγ^+^, CD4 T helper-2 cells: CD45^+^/CD4^+^/IL-4^+^, CD4 T helper-17a cells: CD45^+^/CD4^+^/IL-17a^+^, CD4 T helper-22 cells: CD45^+^/CD4^+^/IL-22^+^ and Treg cells: CD45^+^/CD4^+^/CD25^+^/FoxP3^+^.

### Real time PCR analysis

Groups of mice were sacrificed at 12 weeks post-infection. Lungs were removed and homogenized as described above and total RNA was obtained using TRIzol^®^ (Invitrogen, Carlsbad, CA, USA) and treating samples with DNase I (Thermo Fisher Scientific Inc, Waltham, MA, USA). cDNA was synthesized using 500ng of total RNA using the Maxima First Strand cDNA synthesis kit for RT-qPCR according to the manufacturer’s instructions (Thermo Fisher Scientific Inc, Waltham, MA, USA). Real-time PCR was done using Maxima SYBR Green/Fluorescein qPCR Master Mix (2X) according to the manufacturer’s instructions (Thermo Fisher Scientific Inc, Waltham, MA USA). The CFX96 Real-Time PCR Detection System (Bio-Rad, Headquarters Hercules, California, USA) was employed to measure gene expression levels. Melting curve analysis was performed after the amplification phase of real time PCR assays to eliminate the possibility of non-specific amplification or primer-dimer formation. Validation of housekeeping genes for normalization mRNA expression was performed before gene expression analysis. Fold changes in target gene mRNA expression were quantified relative to Glyceraldehyde-3-phosphate dehydrogenase (*GAPDH*; housekeeping gene previously defined). Each experiment was conducted in duplicate and the expression level measured in triplicate. Primers sequences are listed in Table 1.

**Table 1:** Primers sequences.

Gene	Primer forward (5´—3´)	Primer reverse (5´—3´)
GAPDH	CATGGCCTTCCGTGTTCCTA	GCGGCACGTCAGATCCA
18S RNA	GGTCGGCGTCCCCCAACTTCT	CGTGCAGCCCCGGACATCTAA
β2-microglobulin	AAGTATACTCACGCCACCCACCG	TTTTTTCCCGTTCTTCAGCATTTG
Collagen-1α2	CGCTCCCAGCCTTCACTCAGAC	CACGTGCGAGCAGGGTTCTTTC
Collagen-3α1	GGATGCAGCCACCTTGGTCAGT	GGCAGTCTAGTGGCTCCTCATCAC
TGF-β1	TACTGCCGCTTCTGCTCCCACTCC	TCGATGCGCTTCCGTTTCACCAG
TGF-β3	GGGGCGTCTCAAGAAGCAAAAG	GGGCCCTCTTCTTCCTCTGACTG
TGF-βRc2	CCGGGGCATCGCTCATCTC	CACACAGGCAACAGGTCAAGTCGT
Tissue inhibitor metalloproteinase-1 (TIMP-1)	GCTAAAAGGATTCAAGGCTGTGGG	CTCTTCACTGCGGTTCTGGGACTT
TIMP-2	CCTCTGGATGGACTGGGTCAC	CGGGTCCTCGATGTCAAGAAACTC
Matrix metalloproteinase-1a (MMP-1a)	TGCCAAAAGCAGTTGTGGAAGATG	GTGGAAGGAGAGCACAATATCGCC
MMP-8	GCCACGATGGTTGCAGAGAAGC	CACTCCACATCGAGGCATTTCCA
MMP-12	GGGCTAGAAGCAACTGGGCAACTG	CCATCTTGACCTCTGGGGCACTG
MMP-13	CCACAGTTGACAGGCTCCGAGAA	GTATTCACCCACATCAGGCACTCC
MMP-14	CGATGAGGGGACTGAGGAGGAGAC	CAGCAGTAGTACCGGCAGGACCAC
MMP-15	AACGCGGGTGACAAGGATGAGG	CCAGGCCCAGAATACAGAGCAACA
CXCR-4	CGATAGCCTGTGGATGGTGGTGTT	TCTGGTGGCCCTTGGAGTGTGAC
CXCL-12	TCCCCATTCTCCTCATCCTCATCT	CTCCCTGCAAAGCCACCGTCTAT
T-bet	CCGGGCCTGGCGAGTTCTT	GCGGGCAATGCGTAGTCCTC
GATA-3	GGTCGGCCAGGCAAGATGAGAAA	TAATGGGGTGGTGGGCTGAGGAT
RORc	CGCCGCAAGCCAGCAGTGTAAT	TCGGGACATGCCCAGAGCCA
Ahr	GGCTGCCAGGCAGGGTTGTGA	GCTGGCCGCGGAGGAGAAAC
FoxP3	CCAACGCCCCAACAAGTGCTC	TCATCGCCCGGTTTCCATAGGTA
Inducible Nitric oxide Synthase-2 (iNOS2)	GCCGCATGAGCTTGGTGTTTG	GCAGCCGGGAGTAGCCTGTGT
Arginase-1	CCTTGGCTTGCTTCGGAACTCA	CTTGGGAGGAGAAGGCGTTTGC

### Histopathological analysis

Before detaching the tissue from the mouse, lungs were perfused using 10ml of 1X PBS in order to wash out red blood cells followed by 10ml of formalin (4% Formaldehyde solution, EM Science, Gibbstown, NJ), 0.15M sodium dihydrogen phosphate (Merck, Darmstadt, Germany), and 0,11M sodium hydroxide (Sigma-Aldrich, Saint Louis, MO, USA) to fix the tissue. Lungs were removed and submerged in 4% formalin until ready to be processed. Fixed tissues were embedded in paraffin, cut and stained with Hematoxylin and eosin (H&E) in order to determine the lung inflammatory response, methenamine silver to identify *P*. *brasiliensis* yeast cells and Masson’s trichrome to differentiate collagen fibers. Sliced and stained tissues were analyzed using a Nikon Eclipse C*i*-L microscope (Nikon Instruments Inc., Melville, USA) and acquired using a Nikon DS-Fi2 digital camera and NIS Elements 4.30.02 Laboratory Image Software (Nikon Instruments Inc., Melville, USA). Image processing and quantitative analysis of inflammatory response and granulomatous cellular infiltrate by total pulmonary area was performed using ImageJ Software (National Institutes of Health, NIH, Maryland, USA). The percentage of injured area was calculated by dividing the total injured area, which includes cellular infiltrates and inflammatory lesions, by the total area of lung. The average size of the granuloma was obtained from each section of lung tissue per experimental group.

### Total collagen

Groups of mice were sacrificed at 12 weeks post-infection and lungs were removed and homogenized as describe above. Isolation and concentration of total collagen was performed using the homogenized suspension with acid neutralizing reagent (0.5M acetic acid, 0.1 mg/ml pepsin) (Sigma-Aldrich, Saint Louis, MO, USA) and collagen isolation & concentration reagent (Biocolor, Northern Ireland, U.K.) and centrifuged at 12,000 rpm, 4°C, 10 min. Then, the pellets were resuspended with Sircol Dye reagent (Biocolor, Northern Ireland, U.K.) and proceed with the Sircol Soluble Collagen Assay according to the manufacturer instructions.

### Statistical analysis

Data analysis was performed using Graph Pad Prism software version 5 (GraphPad Software, Inc., La Jolla, CA, USA). Medians and IQR were used to analyze fungal load, flow cytometry, cytokines, and chemokines levels. The Mann-Whitney test was used for comparisons between groups in all developed methodologies. Values of *P*<*0*.*05* were considered to be significant.

## Results

### Neutrophils were efficiently depleted using the specific mAb anti-Ly6G during the chronic stages of PCM

Previous studies in our lab showed that the mAb anti-Ly6G (clone 1A8) depleted specifically neutrophils in BALB/c mice inoculated with PBS or 1,5x10^6^ *P*. *brasiliensis* yeasts cells at different early stages (48 and 96h, see Results in [12]).

**Fig 1:**
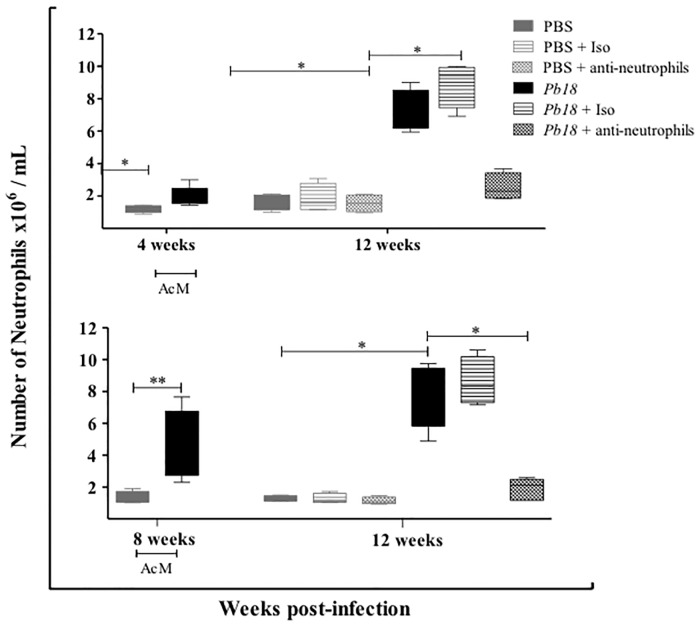
The anti-Ly6G mAb efficiently depleted neutrophils during chronic stage of PCM. BALB/c mice were intranasally inoculated with PBS or 1.5x10^6^ *P*. *brasiliensis* (Pb18) yeast cells, treated with an isotype control Ab or the anti-Ly6G specific mAb against neutrophils during the chronic phase of *P*. *brasiliensis* infection (4 or 8 week post-challenge). Neutrophils were assessed by flow cytometry as described in the Materials and Methods section. Data shown represent median and IQR (n = 4–5 mice/group; representative of two independent experiments). *, P<0.05 and **, P<0.01 comparing infected-untreated mice versus control mice or comparing infected-anti-Ly6G mAb-treated mice versus infected-untreated mice. PBS, control mice; PBS + Iso, control mice treated with isotype control Ab (clone: 2A3); PBS + anti-neutrophils, control mice treated with anti-Ly6G mAb (clone: 1A8); *Pb18*, infected-untreated mice; *Pb18* + Iso, infected mice treated with isotype control Ab; *Pb18* + anti-neutrophils, infected mice treated with anti-Ly6G mAb.

In order to investigate the efficiency of the treatment to deplete neutrophils during the chronic stages of experimental pulmonary PCM, a flow cytometry assay was carried out for lung homogenates at 12 weeks post-infection. We observed that the number of neutrophils remained significantly fewer in infected mice treated with the specific mAb anti-Ly6G, starting such treatment at week 4 or 8 post-infection and analyzed later (12 weeks post-infection), when compared to control mice (Fig 1).

### Depletion of neutrophils was associated with a decreased number of CD4 T cells, B cells, eosinophils, DCs and MDSc in lungs of mice at the chronic stage of the PCM

**Fig 2:**
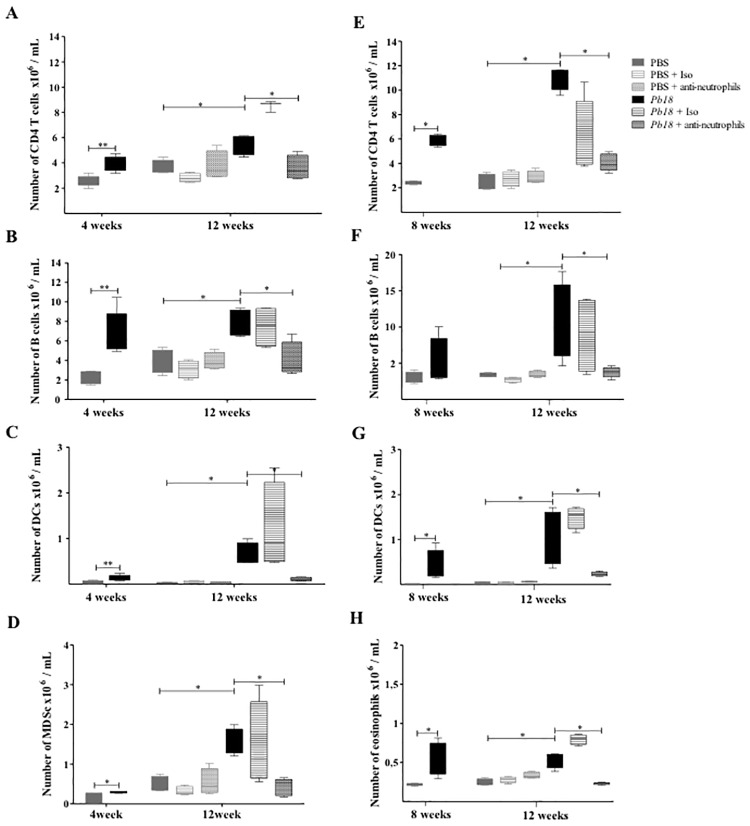
Depletion of neutrophils is associated with a decreased number of CD4 T cells, B cells, eosinophils, DCs and MDSc during the chronic stages of *P*. *brasiliensis* infection. BALB/c mice were inoculated with PBS or 1.5x10^6^ *P*. *brasiliensis* (*Pb18*) yeast cells and treated with an isotype control Ab or anti-Ly6G mAb at 4 weeks (**A, B, C, D**) or 8 weeks (**E, F, G, H**) post-challenge and analyzed at 12 weeks post-infection. Cell populations from lungs of mice were assessed by flow cytometry. CD4 T cells (**A, E**), B cells (**B, F**) DCs (**C, G**), myeloid suppressor cells (**D**) and eosinophils (**H**) were identified as described in the Materials and Methods section. Data shown represent median and IQR (n = 4–5 mice/group; representative of two independent experiments). *, P<0.05; comparing infected untreated mice versus control mice, or comparing infected-anti-Ly6G mAb treated mice versus infected-untreated mice. PBS, control mice; PBS + Iso, control mice treated with isotype control Ab (clone: 2A3); PBS + anti-neutrophils, control mice treated with anti-Ly6G mAb (clone: 1A8); *Pb18*, infected-untreated mice; *Pb18* + Iso, infected mice treated with isotype control Ab; *Pb18* + anti-neutrophils, infected mice treated with anti-Ly6G mAb.

During the chronic stage of the disease, populations of cells were assessed by flow cytometry as described in Materials and Methods. Interestingly, the number of CD4 T cells, B cells, dendritic cells (DCs) and myeloid-derived suppressor cells (MDSc) were significantly lower in the neutrophil-depleted mice treated at week 4 post-infection and analyzed at 12 weeks post-infection compared to untreated or isotype-treated mice (Fig 2A–2D). When the mAb treatment started at 8 weeks post-infection, numbers of CD4 T cells, B cells, DCs and eosinophils also remained significantly lower in those animals compared to control mice at 12 weeks post-infection (Fig 2E–2H).

### Neutrophil depletion is associated with low levels of pro-inflammatory cytokines and chemokines in lungs of mice during the chronic stages of PCM

**Fig 3:**
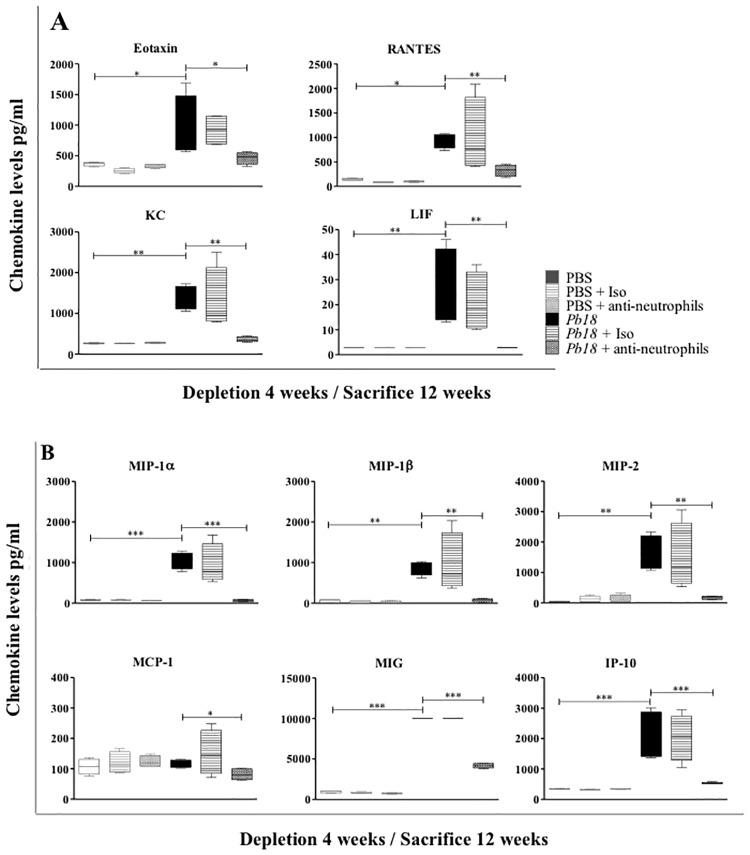
Effect of neutrophil depletion on chemokine levels in lungs during the chronic stage of *P*. *brasiliensis* infection. BALB/c mice were infected with 1.5x10^6^ *P*. *brasiliensis* (Pb18) yeast cells and treated with an isotype control Ab or anti-Ly6G mAb at 4 weeks post-challenge and analyzed at 12 weeks post-infection. Chemokines levels from lungs of mice were assessed by Luminex 200 system as described in the Materials and Methods section. **A**) Chemokines associated with the recruitment of granulocyte cells (neutrophils, eosinophils and basophils); **B**) chemokines associated with the recruitment of mononuclear cells (lymphocytes, monocyte/macrophage and dendritic cells). Data shown represent median and IQR (n = 4–5 mice/group; representative of two independent experiments). *, P<0.05, **, P<0.01 and ***, P<0.001 comparing infected untreated mice versus control mice, or comparing infected-anti-Ly6G mAb treated mice versus infected-untreated mice. PBS, control mice; PBS + Iso, control mice treated with isotype control Ab (clone: 2A3); PBS + anti-neutrophils, control mice treated with anti-Ly6G mAb (clone: 1A8); *Pb18*, infected-untreated mice; *Pb18* + Iso, infected mice treated with isotype control Ab; *Pb18* + anti-neutrophils, infected mice treated with anti-Ly6G mAb.

**Fig 4:**
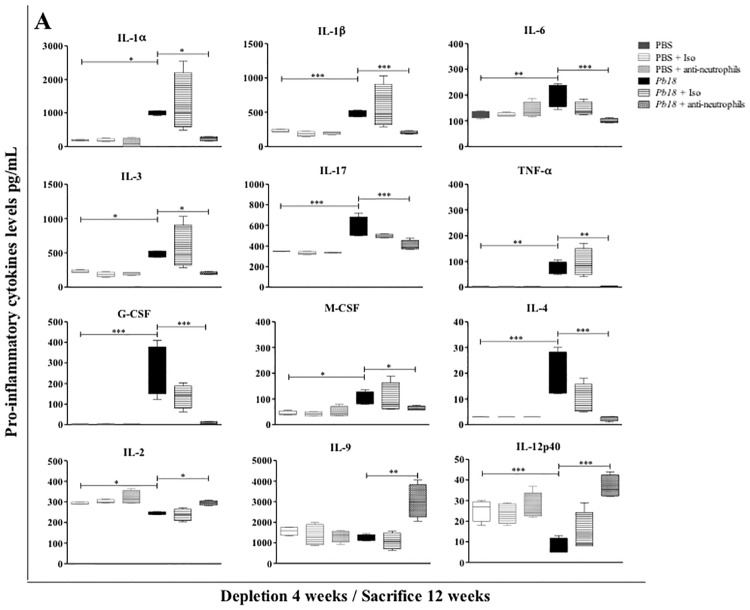
Effect of neutrophils depletion on cytokine levels in lungs during the chronic stage of *P*. *brasiliensis* infection. BALB/c mice were infected with 1.5x10^6^ *P*. *brasiliensis* (Pb18) yeast cells and treated with an isotype control Ab or anti-Ly6G mAb at 4 weeks post-challenge and analyzed at 12 weeks post-infection. Cytokines levels associated with inflammatory response from lungs of mice were measured by Luminex 200 system as described in the Materials and Methods section. Data shown represent median and IQR (n = 4–5 mice/group; representative of two independent experiments). *, P<0.05, **, P<0.01 and ***, P<0.001 comparing infected untreated mice versus control mice, or comparing infected-anti-Ly6G mAb treated mice versus infected-untreated mice. PBS, control mice; PBS + Iso, control mice treated with isotype control Ab (clone: 2A3); PBS + anti-neutrophils, control mice treated with anti-Ly6G mAb (clone: 1A8); *Pb18*, infected-untreated mice; *Pb18* + Iso, infected mice treated with isotype control Ab; *Pb18* + anti-neutrophils, infected mice treated with anti-Ly6G mAb.

Regarding cytokine and chemokine profiles, mice in which treatment with the mAb against neutrophils started at 4 weeks post-infection and evaluated at 12 weeks post-infection showed a significant decrease in levels of chemokines such as CCL11 (eotaxin), CCL5 (RANTES), CXCL1 (KC), LIF, CCL3 (MIP-1α), CCL4 (MIP-1β), CXCL2 (MIP-2), CCL2 (MCP-1), CXCL9 (MIG) and CXCL10 (IP-10) (Fig 3A and 3B), as well as most of the pro-inflammatory cytokines evaluated: IL-1α, IL-1β, IL-3, IL-4, IL-6, IL-17, G-CSF, M-CSF and TNF-α (Fig 4A), when compared to control mice. In addition, a significant increase was observed in levels of IL-2, IL-9 and IL-12p40 in the same group of mice (Fig 4B). When mice were depleted of neutrophils at 8 weeks post-infection and evaluated at 12 weeks post-infection, they showed significant decrease in levels of CCL3 (MIP-1α), CCL4 (MIP-1β), CXCL1 (KC), LIF, G-CSF and M-CSF (Data not shown).

### Depletion of neutrophils was correlated with a reduction in parameters related to Th immunity profiles

**Fig 5:**
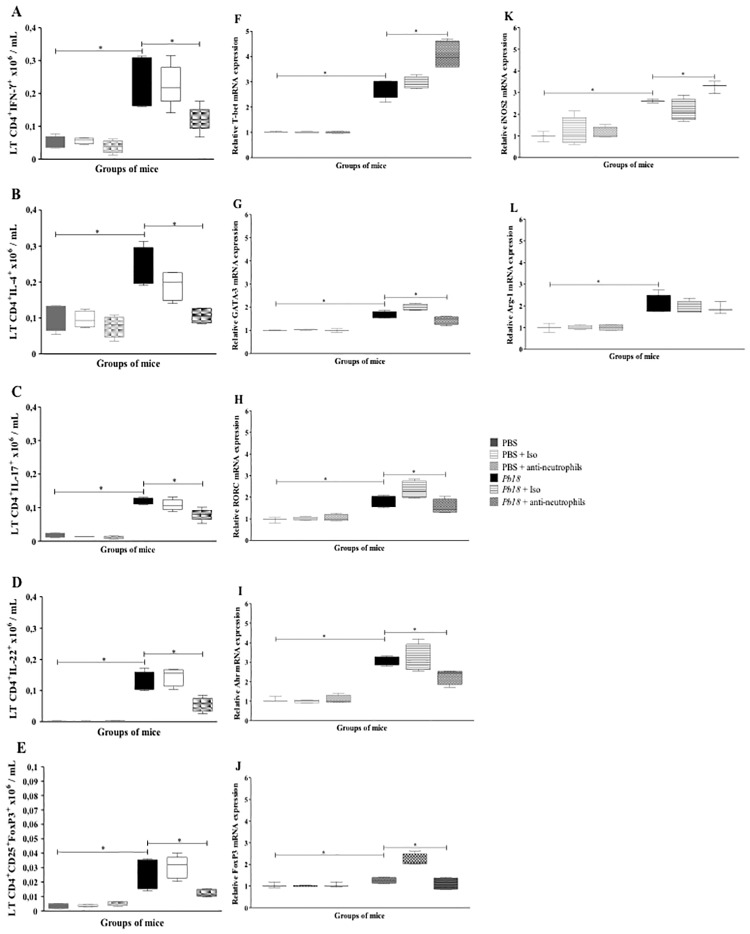
Neutrophil depletion is associated with decreased intracellular cytokine levels and changes in transcription factor mRNA expression related to T helper subsets during the chronic stages of *P*. *brasiliensis* infection. BALB/c mice infected with 1.5x10^6^ *P*. *brasiliensis* yeast cells and treated with an isotype control Ab or anti-Ly6G mAb at 4 weeks post-infected and analyzed at 12 weeks post-infection. Flow cytometry was performed to identify the following lymphocytes subsets: **A**) Th1 (LT CD4^+^IFNγ^+^), **B**) Th2 (LT CD4^+^IL-4^+^), **C**) Th17 (LT CD4^+^IL-17^+^), **D**) Th22 (LT CD4^+^IL-22^+^), and **E**) Treg cells (LT CD4^+^CD25^+^ FoxP3^+^). Data shown represent median and IQR (n = 4–5 mice/group; representative of two independent experiments). Relative quantification of the mRNA expression for T-bet (**F**), GATA-3 (**G**), RORc (**H**), Ahr (**I**), FoxP3 (**J**), iNOS2 (**K**) and Arginase-1 (**L**) were measured in lungs of mice. Results are expressed as mean ± SEM (n = 4–5 mice/group; representative of two independent experiments). *, P<0.05 comparing infected untreated mice versus control mice, or comparing infected-anti-Ly6G mAb treated mice versus infected-untreated mice. PBS, control mice; PBS + Iso, control mice treated with isotype control Ab (clone: 2A3); PBS + anti-neutrophils, control mice treated with anti-Ly6G mAb (clone: 1A8); *Pb18*, infected-untreated mice; *Pb18* + Iso, infected mice treated with isotype control Ab; *Pb18* + anti-neutrophils, infected mice treated with anti-Ly6G mAb.

We evaluated the effect of neutrophils depletion on the Th-related parameters, in terms of cytokines production and transcription factors expression. Overall, *P*. *brasiliensis*-infected mice treated with mAb anti-neutrophils at 4 weeks post-infection showed a decrease in the number of Th1, Th2, Th17, Th22 and Treg subsets population (Fig 5A–5E). Likewise, this neutrophil-depleted group of mice demonstrated a lower mRNA expression of specific transcription factors GATA-3 (Th2), RORc (Th17), Ahr (Th22) and FoxP3 (Treg) (Fig 5G–5J) when compared with infected-isotype control or untreated mice, except for T-bet transcription factor mRNA (Th1) which was found overexpressed in neutrophils-depleted mice (Fig 5F).

We also analyzed the mRNA expression of two other Th1- and Th2-related genes: the inducible nitric oxide synthase-2 (iNOS2) and Arginase-1 (Arg-1), respectively. Our results demonstrated that the iNOS2 mRNA expression was increased in infected and mAb-treated mice when compared to control mice (Fig 5K), meanwhile the Arg-1 mRNA was expressed in similar levels among the different groups of infected mice analyzed (Fig 5L).

### Neutrophil-depleted mice showed a better control of *P*. *brasiliensis* infection during the chronic phase of PCM

**Fig 6:**
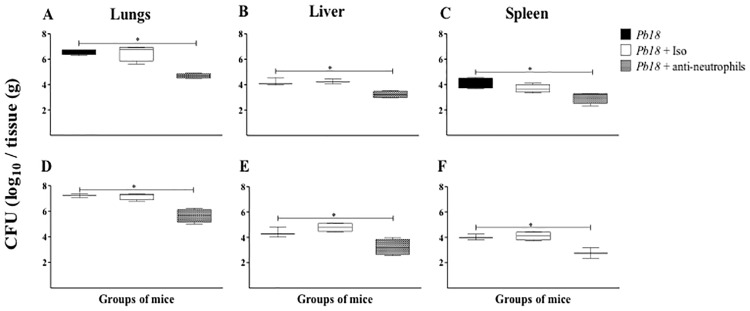
Depletion of neutrophils is associated wit a reduction on the fungal burden in lungs, liver, and spleen from mice infected with *P*. *brasiliensis* at the chronic stages of infection. Fungal load measurement was performed in lung, liver and spleen of mice at 12 weeks post-challenge with 1.5x10^6^ *P*. *brasiliensis* (Pb18) yeast cells and treated with an isotype control Ab or anti-Ly6G mAb at 4 weeks (**A**, **B**, **C**) or 8 weeks (**D**, **E**, **F)** post-challenge. Data shown represent median and IQR (n = 4–5 mice/group; representative of two independent experiments). A statistically significant reduction in fungal burden was observed in the lungs of mice infected and treated with the anti-Ly6G mAb (*, *P<0*.*05*) compared to the infected untreated mice and the infected-isotype control Ab treated mice. *Pb18*, infected, untreated mice; *Pb18* + Iso, infected mice treated with isotype control Ab (clone: 2A3); *Pb18* + anti-neutrophils, infected mice treated with anti-Ly6G mAb (clone: 1A8).

Our next step was to determine the effect of neutrophils depletion on the infection control. The fungal burden was evaluated at 12-week post-infection in lungs, liver and spleen of infected and untreated, isotype control or mAb anti-neutrophils treated mice at 4 or 8-week post-infection. All neutrophil-depleted group of mice showed a significant reduction of fungal burden in all organs assessed compared to infected-untreated or infected-isotype control treated mice (Fig 6A–6F).

### Depletion of neutrophils was associated with reduction of the granulomatous inflammatory response and recovery of pulmonary architecture in mice infected with *P*. *brasiliensis*

**Fig 7:**
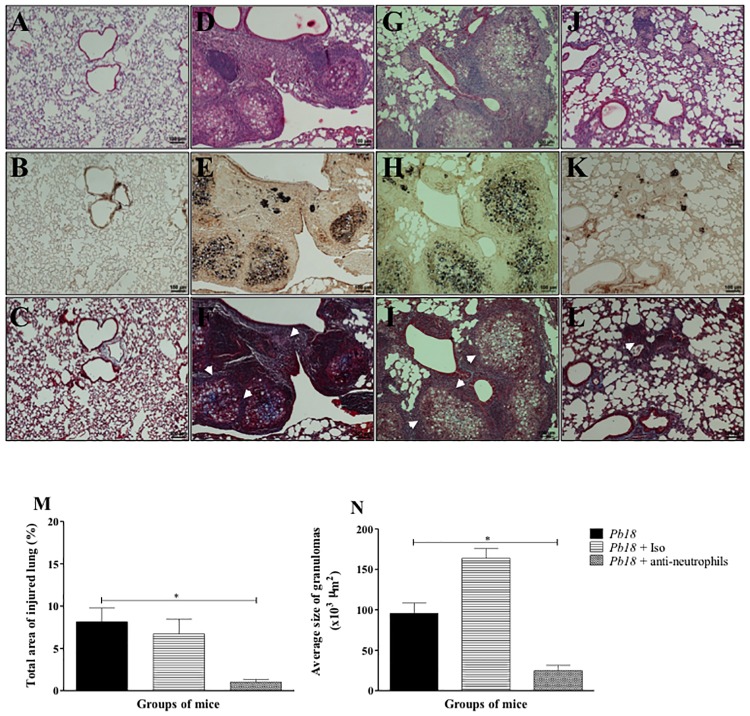
Depletion of neutrophils is associated with a reduction in both granulomatous inflammatory response and fibrosis in lungs from mice challenged with *P*. *brasiliensis*. Microphotographs are representative of lungs from uninfected (**A**, **B**, **C**), infected and untreated mice (**D**, **E**, **F**) or infected and treated with an isotype control Ab (**G**, **H**, **I**) or an anti-Ly6G mAb specific to neutrophils (**J**, **K**, **L**) at 12 weeks post-challenge and obtained from groups of 4–5 mice. Lungs were fixed, embedded in paraffin, cut and stained using H&E staining to determine lung inflammatory response (**A**, **D**, **G**, **J**), methenamine silver staining (**B**, **E**, **H**, **K**) to identify *P*. *brasiliensis’* yeast cells and Masson’s trichrome (**C**, **F**, **I**, **L**) to identify and differentiate collagen fibers as described in the Materials and Methods section. Arrowheads indicate collagen fibers (in white). Quantitative analysis of total injured lung area (**M**) and size of granuloma (**N**) were assessed by ImageJ software as described in the Materials and Methods section. These results are representative of two independent experiments. Magnification 10X.

In order to evaluate the effect of neutrophil-depletion strategy in the lung parenchyma (*in situ*), lungs were processed for histopathological analysis as described in the Materials and Methods section. It was found a granulomatous cellular infiltrate (Fig 7D and 7G) with abundant parasitic yeast form surrounded by collagen fibers (Fig 7E, 7H, 7F and 7I) in the untreated- or isotype treated- and infected mice groups. Meanwhile, infected and treated mice with the anti-neutrophils mAb at 4 weeks post-infection evidenced an apparent decrease in the total area of injured lung (Fig 7M), as well as reduction in the size of granulomas (Fig 7N) and composition of cellular infiltrates (Fig 7J), accompanied by a significant decrease in the amount of *P*. *brasiliensis* yeasts and collagen fibers (Fig 7K and 7L).

### Recovery of the pulmonary architecture was associated to low level of total soluble collagen, an overexpression of collagen-1 mRNA and a decrease expression of collagen-3 gene

**Fig 8:**
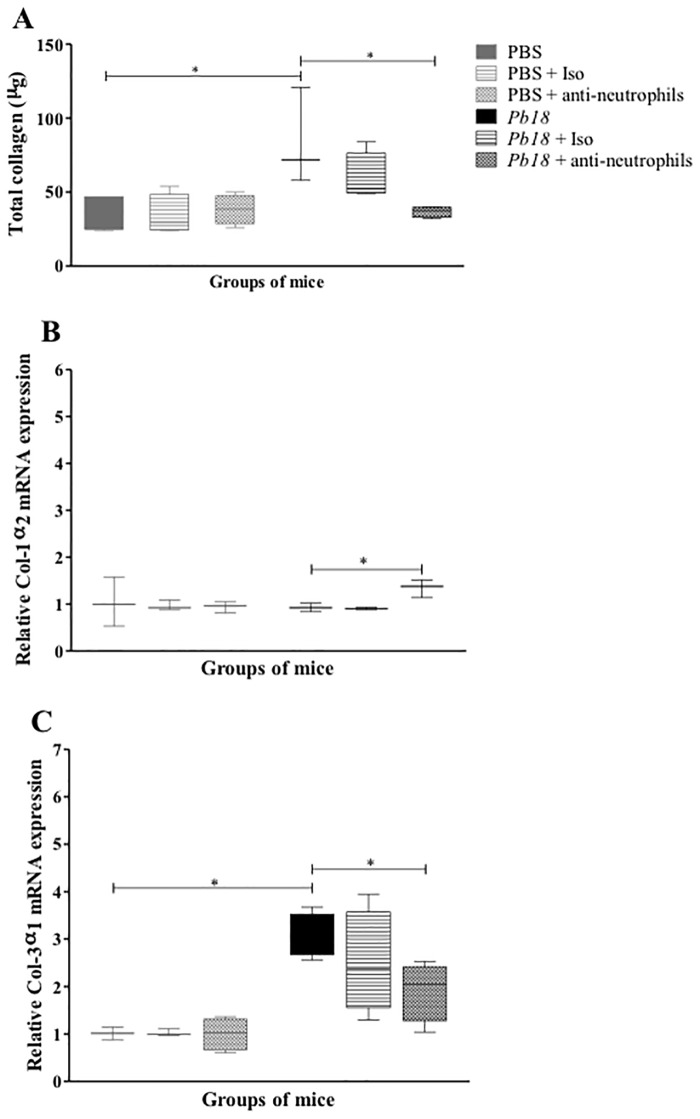
Neutrophils depletion is associated with a decrease of collagen levels and changes on mRNA level expression of Colagen-1 and Colagen-3 in lungs of mice infected with *P*. *brasiliensis* yeast cells. BALB/c mice were infected with 1.5x10^6^ *P*. *brasiliensis* (Pb18) yeast cells and treated with an isotype control Ab or anti-Ly6G mAb at 4 weeks post-challenge and analyzed at 12 weeks post-infection. Total collagen (A) and relative quantification of the mRNA expression for Col-1α2 (B) and Col-3α1 (C) were performed in lungs of mice (n = 4–5 mice/group; representative of two independent experiments). Results are expressed as median ± IQR. *, P<0.05 comparing infected untreated mice versus control mice, or comparing infected-anti-Ly6G mAb treated mice versus infected untreated mice. PBS, control mice; PBS + Iso, control mice treated with isotype control Ab (clone: 2A3); PBS + anti-neutrophils, control mice treated with anti-Ly6G mAb (clone: 1A8); *Pb18*, infected, untreated mice; *Pb18* + Iso, infected mice treated with isotype control Ab; *Pb18* + anti-neutrophils, infected mice treated with anti-Ly6G mAb.

In order to determine if the collagen, considered as the most important extracellular matrix protein (ECMp) involved in the fibrosis development, was associated with the controlled granulomatous inflammatory process and the recovery of pulmonary architecture in *P*. *brasiliensis*-infected mice, we determined the production and expression of this ECMp *in situ*. It was observed that the total amount of soluble collagen was diminished in neutrophils-depleted mice (Fig 8A) compared to control animals. Interestingly, neutrophil-depleted mice showed an increase expression of collagen-I and a decrease expression of collagen-III mRNA (Fig 8B–8C).

### Reduction of granulomatous inflammatory response in lungs of neutrophil-depleted and infected mice was associated with an altered expression pulmonary fibrosis-related genes: TGF-β, MMP and TIMP

**Fig 9:**
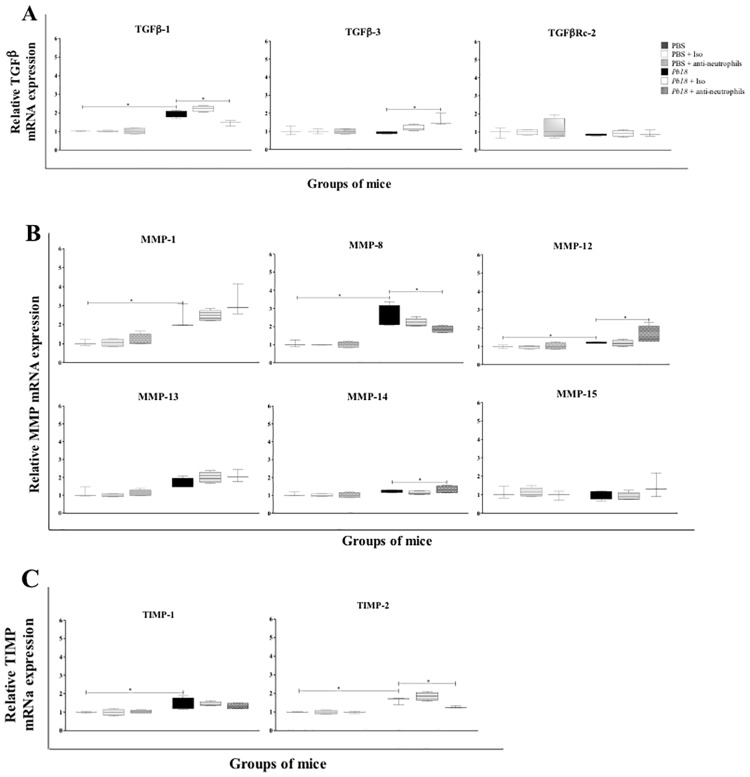
Reduction of the granulomatous inflammatory response in lungs of neutrophil-depleted mice challenged with *P*. *brasiliensis* during the chronic course of infection is associated with a decrease of expression of TGF-β1 and MMP-8 and overexpression of TGF-β3, MMP-12 and MMP-14. BALB/c mice were infected with 1.5x10^6^ *P*. *brasiliensis* (Pb18) yeast cells and treated with an isotype control Ab or anti-Ly6G mAb at 4 weeks post-challenge and analyzed at 12 weeks post-infection. Relative quantification of the mRNA expression was performed in lungs of mice for the following genes: **A**) TGF-β1, TGF-β3 and TGF-βRc2; **B**) MMP-1, MMP-8, MMP-12, MMP-13, MMP-14 and MMP-15; and **C**) TIMP-1 and TIMP-2 (n = 4–5 mice/group; representative of two independent experiments). Results are expressed as median ± IQR. *, P<0.05 comparing infected untreated mice versus control mice, or comparing infected-anti-Ly6G mAb treated mice versus infected untreated mice. PBS, control mice; PBS + Iso, control mice treated with isotype control Ab (clone: 2A3); PBS + anti-neutrophils, control mice treated with anti-Ly6G mAb (clone: 1A8); *Pb18*, infected, untreated mice; *Pb18* + Iso, infected mice treated with isotype control Ab; *Pb18* + anti-neutrophils, infected mice treated with anti-Ly6G mAb.

Finally, we evaluated the effect of neutrophils-depletion treatment on the mRNA expression of genes associated with the development of pulmonary fibrosis. Our results demonstrated that the mRNA expression of TGF-β1, Matrix metalloproteinase (MMP)-8 and Tissue inhibitor metalloproteinase (TIMP)-2 genes were significantly diminished in infected and mAb treated-animals compared to control ones (Fig 9). In contrast, the mRNA of TGF-β3, MMP-12 and MMP-14 genes were found overexpressed in this group of mice (Fig 9).

## Discussion

Several studies have been highlighting the importance of neutrophils in the pathogenesis of PCM, most of them dedicated to evaluate the role of these cells during early stages of infection and their influence on the adaptive immune response [8,11,12]. However, to our knowledge, there are no studies focused on evaluating neutrophil involvement in the chronic stages or in the development of fibrosis observed in this mycosis. In the present study, we describe for the first time that the depletion of neutrophils during chronic stages of PCM is associated with a better control of *P*. *brasiliensis* infection, reduction in the inflammatory and granulomatous response, and attenuation of the fibrosis process as observed by recovery of the pulmonary architecture with decreased collagen fibers and lower expression of genes associated with development of fibrosis.

Neutrophils are considered pivotal as the first line of innate immunity and as promoter of adaptive immune response that contribute to the development of inflammatory reaction against a wide range of infections [14–16]. Many strategies have been used to understand neutrophil functions during recognition and elimination of a pathogenic microorganism [17–19]. Those strategies include genetic manipulation (*Gfi-1*^*-/-*^ knockout) [20] or the implementation of monoclonal antibodies such as anti-Gr1 (RB6-8C5) or anti-Ly6G (1A8) in animal models inducing depletion of neutrophils [21], the latter being most widely used to evaluate the *in vivo* functions of these cells during the development of immune response against infections caused by *Toxoplasma gondii* [22], *Leishmania amazonensis* [23], and fungal infections caused by *Aspergillus fumigatus* [24], *Cryptococcus neoformans* [25,26] and *Candida albicans* [27]. In the case of *P*. *brasiliensis* infection, a study involving resistant (A/J) or susceptible (B10.A) mice to fungus and treated with the anti-Gr1 mAb during the early stages of infection showed that neutrophils exert a protective role on the innate phase of immunity only in the susceptible host infected with the fungus [11]. Similar results were obtained in our laboratory using a new experimental PCM model in BALB/c mice infected i.n. with *P*. *brasiliensis* yeast and treated with the specific anti-neutrophils mAb (anty-Ly6G; clone 1A8) during the acute phase of infection, demonstrating a decrease in survival time complemented with an increased fungal burden and exacerbation of the inflammatory response [12].

The goal of the present study was to evaluate the effect of neutrophils depletion during the chronic stage of PCM and specially during the development of fibrosis process; herein, we demonstrated that depletion of these phagocytic cells was associated with a better control of infection, as shown by lower fungal burden in their lungs, livers, and spleens, suggesting that the presence of neutrophils may be contributing to the persistence of *P*. *brasiliensis*. Several *in vitro* studies have described the interaction between *P*. *brasiliensis* yeasts and neutrophils hypothesizing about how this phagocytic cell promotes the replication of fungus allowing the infection to be spread from lungs to other organs and systems. Kurita *et al* [28–30] suggested that *P*. *brasiliensis* yeast could evade the immune response and survive inside the neutrophil. In addition, Acorci *et al* [31] reported that the ability of yeast cells to inhibit apoptosis and prolong the lifetime of neutrophils via IL-8. Moreover, it has been demonstrated that neutrophils from patients with PCM have a significant deficiency to digest *in vitro* viable *P*. *brasiliensis* yeasts without prior activation by IFN-γ, GM-CSF and/or IL-1β [32,33].

Likewise, our findings may indicate that the absence of neutrophils promotes the development of an effective immune response leading to reduced tissue damage. Similar results have been reported in an animal model of *C*. *neoformans* infection, in which it was found that neutrophils depletion increased levels of pro- and anti-inflammatory cytokines, favoring the infection control [34]. Additionally, Park *et al* [35] using an animal model of neutropenic invasive aspergillosis postulated that the absence of neutrophils in infected tissue enhances the inflammatory cytokine milieu and cellular response to *Aspergillus* in the host. Similarly, Dunay *et al* [22] observed that *T*. *gondii*-infected mice treated with the anti-neutrophils mAb were able to control the parasite replication, increase their survival to infection accompanied by a reduction in number of CD4 and CD8 T cells. Interestingly, in the present study we observed that neutrophil depletion in *P*. *brasiliensis*-infected mice was associated with diminished number of CD4 T, B cells, DCs, and Treg cells as well as pro-inflammatory cytokines and chemokines. Thus, these results confirm that neutrophils play an important immunoregulatory role in the pathogenesis of PCM.

On this line, several authors have demonstrated the ability of neutrophils to regulate leukocyte trafficking through multiple chemokine production [36,37]. In our case, this phenomenon could be linked to the significant reduction in the levels of several chemokines in infected mAb-treated mice, which are involved in the recruitment of mononuclear and polimorphonuclear cells (Fig 3) [38,39]. It is also clear that neutrophils produce a large number of cytokines during infection [40,41], many of which, in our case, were found significantly decreased in neutrophil-depleted mice. Chen *et al* [42] observed from a cerebral malaria animal model, that in the absence of neutrophils the expression of pro-inflammatory cytokines and the development of cerebral microhaemorrhages were reduced, supporting the hypothesis that this phagocytic cell plays a critical role in coordinating the inflammatory response and is determinant in the pathogenesis of malaria infection.

Regarding cell populations, our results showed a lower number in eosinophils and MDSc on neutrophil-depleted and infected mice compared to control animals. Some authors have associated these populations of cells with a Th2-profile [43,44] and suppression of adaptive immunity [45] that could promote the development of severe forms of PCM [46,47]. Of note, eosinophilia has been associated with worsening and a poor prognosis of fungal infections including PCM and coccidioidomycosis, a process linked to a Th2 immunity pattern, which is less efficient to control infection; additionally, the toxic radical molecules stored in their granules could be also associated to the tissue damage observed [43,48–51]. On the other hand, MDSc are characterized by their capacity to suppress T cell response and subsequently modulate the outcome of fungal infections [52]. Thus, in the present study, depletion of neutrophils was associated with a diminished number in eosinophils and MDSc accompanied by a reduction on fungal burden and an improvement of control infection as well as of recovery of damaged tissue.

Furthermore, some reports have evidenced that resistance to *P*. *brasiliensis* infection depends mainly upon Th1 and Th17 cells [46,53,54]. However, in some cases, this T cell response can lead to massive destruction of lung tissue affecting its functionality, as seen in patients with PCM and fibrosis [1,6]. Likewise, it has been suggested the importance of neutrophils in adaptive immunity and pulmonary host defenses. Loures *et al* [55] showed that Toll-like receptor (TLR)-2-deficient mice infected with *P*. *brasiliensis* yeasts develop a prevalent Th17 immunity with a diminished presence of Tregs, allowing control fungal growth but also inducing tissue damage mediated by neutrophils. Later on, the same group reported that TLR4-deficient mice were unable to control the infection and developed an inefficient immune response by decreased expansion of Th17 cells and elevated differentiation of Treg, which could negatively control the expansion and migration of *P*. *brasiliensis-*specific T cells to the lungs [56]. In contrast, our findings showed that neutrophil-depletion treatment was associated with a reduction of CD4 T cell subsets, including Th17 and Tregs accompanied by higher levels of IL-2, IL-9 and IL-12 cytokines and the overexpression of T-bet and iNOS genes, thus supporting our hypothesis of the development of an effective immune response that controls not only the fungal growth but also the intensity of the tissue damage.

Finally, regarding pulmonary fibrosis induced by *P*. *brasiliensis* infection, previous reports obtained from animal models have described the formation of granulomatous infiltrates starting at 4 weeks post-infection [57,58] and increased amounts of collagen, TGF-β and TNF- α levels [59,60] as observed in the present work. Interestingly, after treatment with the anti-neutrophils mAb, we observed a decrease in the amount of collagen, the inflammatory area, the size of granulomas, the number of Tregs, and the expression of TGF-β1. This might be explained by an indirect mechanism in which the neutrophil produces IL-17 a pivotal cytokine that subsequently induces the recruitment and activation of Treg cells, as well as the induction of the TGF-β production by type II macrophages which in turn activate fibroblast to produce collagen. Thus, we previously demonstrated that neutrophils are an important source of IL-17 [12]. Moreover, in a previous report it was demonstrated that the usage of a combined itraconazole-pentoxifylline treatment in *P*. *brasiliensis*-infected mice, initiated at 4 and 8 weeks post-infection, reduced both the inflammatory response (including neutrophils) and fibrosis [61]; herein, we confirmed that reduction of the fibrosis process was accompanied by a lower number of neutrophils as well as IL-17, Th-17, and Treg cells.

An additional hypothesis suggests that neutrophil induces the production and over-activation of proteolytic enzymes, such as MMPs, responsible for extracellular matrix (mainly collagen) remodeling and degradation [62,63]. In pathological conditions, the MMPs are associated with tissue destruction, weakening of extracellular matrix (ECM) and fibrosis [64,65]; once this happens, TIMPs may act in the tissue environment to neutralize the MMP, thereby preventing excessive degradation [62,66]. In the present study, we observed a decrease in mRNA expression of MMP-8 (collagenase-III), known as “neutrophil collagenase” [67,68]. Additionally, those infected and treated mice showed an increased level of collagen-I, TIMP-2, MMP-12 and MMP-14 genes, associated with a significant reduction of the fibrotic sequelae and a tissue remodeling process [69]. Although for many years fibrosis was thought to be a progressive and irreversible process, recent studies have challenged this theory. It is possible that a controlled inflammatory response could facilitate ECM degradation and remodeling [70].

In summary, we demonstrated that neutrophils play a dual role during *P*. *brasiliensis* infection, being protecting during early infection and harmful during the chronic phase of disease ([12] and the present study). Thus, we could hypothesize that during early infection neutrophils interact with macrophages and DCs through production of chemokines and cytokines, which in turn induce their recruitment, activation, differentiation and maturation. In the case of macrophages, the IFN-γ produced by neutrophils [12] and/or other innate cells, activate and enable them to kill the fungus through potent antifungal mechanisms, whereas interactions with DCs could promote their maturation with the subsequent development and expansion of T cell-mediated immunity, as described for *A*. *fumigatus* infection [71]. In contrast, during the chronic phase of fungal infection, the recruitment of neutrophils induces an uncontrolled immune reaction, leading to a severe tissue inflammatory pathology due to the excessive release of IL-17, TNF-α and enzymes contained in their granules [14,72]. Additionally, neutrophils may harbor the fungus, acting as “Trojan horses”, allowing it to survive inside these phagocytic cells and disseminate from the site of infection to other organs and systems, as described for other pathogens [14,73,74]. Moreover, different neutrophil subsets have been described and characterized by the cytokine and chemokine production, and by different surface antigens expression, as well [75]. However, the identification of neutrophil subpopulations has not been described in fungal infections so far. In consequence, it could not be ruled out that different neutrophil subsets act at different phases of fungal infection, leading to an opposite immune response.

## Conclusions

The above results indicate that neutrophil depletion was associated with a better control of infection and decrease of the granulomatous inflammatory response and fibrosis during the chronic stages of experimental PCM, suggesting that this phagocytic cell exert a harmful role in the immune response to *P*. *brasiliensis* infection and could influence the development of fibrosis in affected individuals. Thus, neutrophil could be considered as a possible therapeutic target in PCM.
